# HIV services for fisherfolk in Sierra Leone: A formative assessment of barriers, opportunities, and community preferences

**DOI:** 10.1371/journal.pgph.0005411

**Published:** 2025-11-18

**Authors:** Jennifer M. Zech, Oliver Eleeza, Martin Msukwa, Tsitsi Masvawure, Haja Bah, Henry Sandy, Amara Vandi, Sheka H. Kargbo, Victoria Kamara, Kemoh Mansara, AbdulRaheem Yakubu, Mame Toure, Miriam Rabkin

**Affiliations:** 1 ICAP at Columbia University, Mailman School of Public Health, New York, New York, United States of America; 2 ICAP Sierra Leone, Freetown, Sierra Leone; 3 SEED Global Health, Boston, Massachusetts, United States of America; 4 Department of Integrative and Global Studies, Worcester Polytechnic Institute, Worcester, Massachusetts, United States of America; 5 Ministry of Fisheries and Marine Resources, Freetown, Sierra Leone; 6 National AIDS Control Program at the Ministry of Health, Freetown, Sierra Leone; 7 National HIV and AIDS Secretariat, Freetown, Sierra Leone; 8 Department of Epidemiology, Columbia University Mailman School of Public Health, New York, New York, United States of America; The Aurum Institute, SOUTH AFRICA

## Abstract

In African fishing communities, landing sites are central to social and sexual networks, making them important areas for HIV transmission. Despite being identified as a priority group by the Sierra Leone Ministry of Health (MoH), there are limited data on fisherfolk’s HIV risk or their preferences regarding HIV prevention, testing, linkage and treatment services. In May 2022, we conducted a formative assessment at two landing sites, using surveys and focus group discussions with 113 fisherfolk, including fishermen, fishmongers, fish traders, fish processors and community members, along with 17 interviews with MoH staff, fishermen union consortium, and health providers. Participants were mostly female (56%), married (70%), and middle-aged (median age 40); 64% had only primary education or less. While all had heard of HIV, 69% considered themselves at no or low risk, despite 32% having multiple recent sexual partners, 58% not knowing a partner’s HIV status, and 34% not using condoms in the past month. Nearly half (48%) reported a prior STI, yet only 7% had heard of PrEP. Thirteen participants (12%) self-reported being HIV-positive, but just two were on treatment. Qualitative findings echoed low HIV knowledge and risk perception, with many noting the absence of recent condom distribution or HIV education efforts. Fisherfolk expressed a preference for receiving HIV services from nurses, ideally near landing sites. Stakeholders affirmed the need for targeted interventions. This assessment highlights a disconnect between risk behaviors and risk perception, and a lack of access to essential HIV services. To improve uptake and outcomes, it is critical to engage fisherfolk in designing community-based, differentiated HIV service delivery strategies.

## Introduction

HIV incidence and prevalence are typically higher in fishing communities than in the general population [1–4]. In Africa, as elsewhere, fisherfolk (i.e., fishermen, fishmongers, fish traders, fish processors, and community members engaged in the fishing economy) face unique challenges due to synergistic structural, cultural, social, and economic factors [5–8]. Fishermen unload their catch, sell their products, buy/gather supplies, and store their boats at landing sites on the shore, which serve as the nexus for fishing activities, social interactions, and sexual networks, thus contributing to HIV transmission [9,10]. The inherent mobility of the fisherfolk lifestyle, often involving extended periods away from home, can increase HIV risk by facilitating casual sexual norms, multiple concurrent sexual partnerships, transactional sex, and heavy alcohol use [11,12]. Adherence to HIV treatment for such mobile individuals can also be challenging [13–16].

Sierra Leone, a coastal country in West Africa, has a large and economically significant fishing industry, employing over 500,000 people in the country of 8.9 million. Of these, more than 100,000 are artisanal fishermen operating at 641 landing sites across six major fishing districts where fishing activities are concentrated [17]. In 2022, fisheries contributed 14% to the national GDP, one of the highest in the world [17]. While comprehensive data on HIV prevalence and risk behaviors among Sierra Leonean fisherfolk are limited, small studies suggest HIV-risk patterns similar to those in other regional fishing communities, exacerbated by economic stresses from declining fish stocks [18,19]. There is a dearth of recent data on HIV prevalence among fisherfolk in Sierra Leone. However, in 2011, the HIV Surveillance on Fisherfolks in Sierra Leone report found HIV prevalence of 3.9% amongst fisherfolk, compared to 1.5% in the general population in 2010 [20]. The 2010 Sierra Leone HIV Modes of Transmission Study revealed that in 2008, 10.8% of new HIV infections were among fisherfolk, with an incidence rate of 560/100,000, compared to 213/100,000 in the general population [21].

Recognizing the importance of fisherfolk to Sierra Leone’s economy and their potential role as a bridging population for HIV transmission, the Ministry of Health (MoH) has prioritized fisherfolk in the national HIV response [22]. Embracing the principle of HIV differentiated service delivery [23], MoH now aims to design person-centered HIV prevention, testing, and treatment for key and priority populations, which includes fisherfolk.

In order to support the design of these tailored HIV services, ICAP at Columbia University conducted a mixed-methods formative evaluation with fisherfolk and fishing community members at landing sites in Sierra Leone in collaboration with the National AIDS Control Program (NACP) at MoH, the National HIV and AIDS Secretariat (NAS), the Ministry of Fisheries and Marine Resources (MFMR), and the Artisanal Fishermen Union Consortium (AFUC). The study aimed to assess HIV-related knowledge, attitudes, risk behaviors and preferences for HIV and health services amongst fisherfolk. The results of this exploratory assessment offer valuable insights into the lived experiences, HIV risk factors, access to HIV and other healthcare services, and the health service design and delivery preferences of this underserved and vulnerable population, helping to inform more targeted and effective interventions and future studies.

## Methods

### Ethics statement

The study was approved by Columbia University’s Institutional Review Board (AAAU0100) on March 29, 2022, the Sierra Leone Ethics and Scientific Review Committee, and the U.S Health Resources & Services Administration on February 2, 2022.

### Study setting

Sierra Leone is one of the poorest countries in the world, ranking 185 out of 193 countries in the Human Development Index (HDI), with a GNI per capita of approximately $1714 [24]. The number of people living with HIV in Sierra Leone has steadily increased with HIV prevalence estimated at 1.4 percent among adults aged 15–49 (1.8 and 0.9 percent in women and men respectively) in 2023 [25]. As of 2019, HIV prevalence in urban areas is twice that in rural areas, at 2.3 percent in urban areas compared with 1.2 percent in rural areas. The Western Area region has the highest HIV prevalence (2.5 percent), about twice the level compared with the other regions, and within this region, Western Rural district has the highest HIV prevalence, at 3.4 percent [26]. Despite its limited health budget, Sierra Leone has made some progress towards achieving the 95-95-95 targets, with 83% of people living with HIV aware of their status and 83% of those aware of their status receiving antiretroviral therapy, and no data on treatment achieving viral suppression [25].

**Fig 1:**
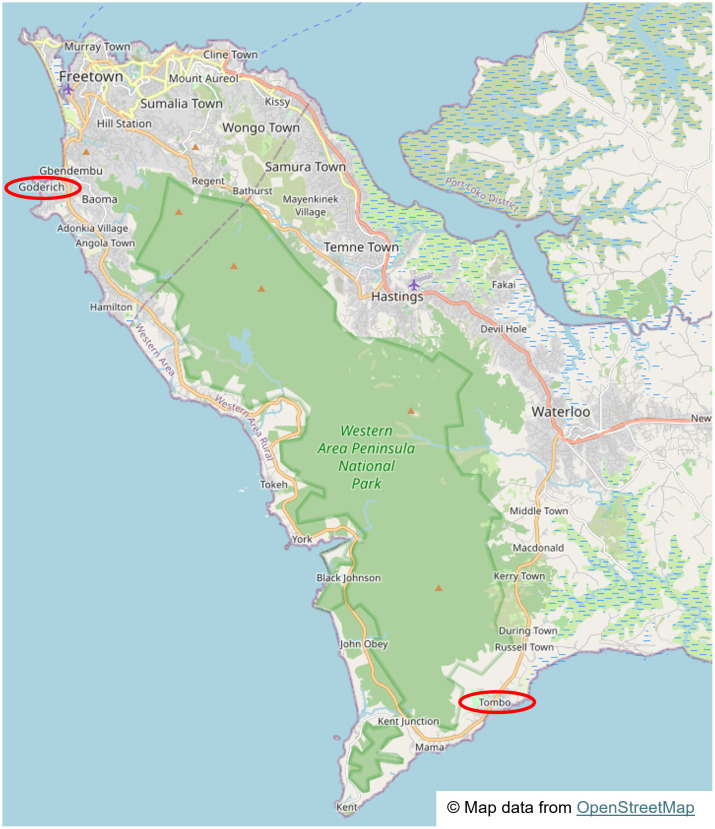
Location of the study sites showing the two fisherfolk landing sites in the Western Area of Sierra Leone. Map data from OpenStreetMap: https://www.openstreetmap.org/copyright Direct map link: https://www.openstreetmap.org/?#map=12/8.3219/-13.2296.

For this study, data were collected at two landing sites, Goderich and Tombo, within the Western Rural district of Sierra Leone. Sites were purposively chosen in high HIV prevalence areas and densely populated landing sites. The landing sites were selected in partnership with NACP, MoHS, NAS, MFMR, and AFUC (Fig 1).

### Study design and sample size

Mixed-methods were used to explore knowledge, attitudes, and preferences for health and HIV services amongst fisherfolk. Data collection took place in May 2022 and included surveys and focus group discussions (FGDs) with fisherfolk as well as key informant interviews (KIIs) with stakeholders. FGD guides and the survey questionnaire were written in English, translated into Krio (Sierra Leone’s most common local language) during administration of the tools, piloted, and revised based on feedback.

FGDs were conducted with fisherfolk to understand the structural and social barriers to health care, knowledge of health and HIV, and current practices concerning health services. We aimed to conduct a total of twelve FGDs, six at each site, (with 6–10 participants per FGD): about one third with fishermen, one third with fishmongers, and one third with people who participate in the fishing communities in additional ways. Surveys were administered to the same fisherfolk who participated in the FGDs, to assess preferences for specific health and HIV program delivery elements.

KIIs were conducted with stakeholders, including health care providers at nearby health facilities, leadership of the artisanal fishermen union consortium, MFMR and MoHS to provide contextual information about perceived healthcare needs and barriers and facilitators to accessing healthcare services, knowledge of health and HIV, and current practices with regards to health services among fisherfolk. KII sample size was designed to attain saturation with a goal of a maximum of 20 KIIs.

### Recruitment and data collection procedures

Prior to data collection, 12 research assistants (6 female; 6 male) with bachelor’s degrees or equivalent or higher and prior experience in conducting qualitative and quantitative research participated in a four-day workshop covering study tools and procedures. Three Fisheries Officers, provided by the MFMR, served as liaisons for the study and participated in the training. Training focused on augmentation of both qualitative (e.g., listening, reflecting, summarizing) and quantitative research skills (e.g., survey administration, data collection programming, data accuracy), research ethics, and included practical sessions where research assistants practiced surveys, KIIs, and FGDs to build familiarity and consistency with the tools.

At each landing site, the study team met with the harbor master to describe the study and the eligible participants: fisherfolk (fishermen, fishmongers, and other members of the fishing community) aged 18 years or older who had been a member of the fishing community for at least one year and spoke English and/or Krio. The harbor masters identified a convenience sample of fisherfolk and referred them to the study team, who met with potential participants to explain the study, invite them to participate, and obtain written informed consent. FGDs were stratified by gender and participant type. FGDs were conducted in Krio; each took approximately 90 minutes. They were audio-recorded, transcribed and translated into English. A bilingual research staff member validated each FGD transcript for completeness and accuracy prior to coding. Surveys were conducted among the same fisherfolk participants from the FGDs.

Semi-structured KIIs were conducted with national and regional level stakeholders identified by purposive followed by snowball sampling. Following written informed consent, research staff conducted in-person interviews in English; each took approximately 50 minutes. Interviews were audio-recorded, transcribed, and reviewed for accuracy and completeness by senior research staff before analysis.

### Data analysis

KIIs and FGDs were entered and analyzed using the Dedoose Software Package (version 8.1.8). A team of two researchers coded each transcript by question and key theme, meeting frequently to compare and reconcile the application of thematic codes. Code reports were analyzed by two researchers who initially worked independently and then met to share and discuss their initial impressions about participants’ views on health and HIV services. These findings were condensed into summaries and shared with other study team members. The researchers conducted framework analysis [27] to organize the data by theme and a final coding scheme was achieved through consensus discussions among the research team [28].

Survey data were transferred from the cloud-based server to the SPSS statistical software package, where entries were error-checked and summary variables created to produce a final analytic dataset. Descriptive analyses were conducted using SPSS. Tests of significance were conducted using Chi-square or Fisher’s Exact tests for categorical variables and t-tests for continuous variables. We examined characteristics according to sex; p-values <0.05 and risk ratios (RR) with a 95% confidence interval (CI) are noted in the tables and footnotes for categorical variables.

## Results

We conducted 12 FGDs and 113 surveys with 113 fisherfolk, and 17 KIIs with stakeholders.

### Participant demographics

The 113 fisherfolk (55 from Goderich and 58 from Tombo) included 37 fishermen, 38 fishmongers, 9 fish processors, 15 sex workers, and 14 other professions; 56% were female and the median age was 40 years. Most fisherfolk (64%) had a primary education or less (26% primary, 38% less than primary), while 35% had a secondary education and only 1% had education beyond secondary school. Almost half of the fisherfolk (49%) were married monogamously, 21% were in polygamous marriages, and 20% had never married. The remainder were widowed (8%), divorced or separated (1%), or living with a partner without being married (1%). Fisherfolk reported having lived in their fishing community for a median of 21 years. Participants were highly mobile, with 20% reporting being away from the community for more than one month at a time in the past year. Fishermen and sex workers were the most likely to have been away from the community for more than one month (Table 1).

**Table 1:** Fisherfolk participant demographics, N = 113.

	Fishermen (N = 37)		Fishmongers (N = 38)		Fish Processors (N = 9)		Sex workers (N = 15)		Other profession (N = 14)		TOTAL (N = 113)	
	n	%	n	%	n	%	n	%	n	%	n	%
**Gender (% female)**	0	0	34	90	2	22	15	100	12	86	63	56
**Age (median, range); N = 111**	43 (25-64)		45 (27-70)		40 (25-53)		23.5 (18-35)		38 (18-60)		40 (18-70)	
**Highest level of education**												
None	7	19	19	50	5	56	4	27	8	57	43	38
Primary	16	43	7	18	2	22	0	0	4	29	29	26
Secondary	14	38	11	29	2	22	11	73	2	14	40	35
More than secondary	0	0	1	3	0	0	0	0	0	0	1	1
**Marital status**												
Single (never married)	5	14	3	8	0	0	13	87	2	14	23	20
Married monogamous	23	62	19	50	6	67	1	7	6	43	55	49
Married polygamous	8	22	11	29	1	11	0	0	4	29	24	21
Living with partner, unmarried	0	0	0	0	0	0	0	0	1	7	1	1
Divorced/separated	1	3	0	0	0	0	0	0	0	0	1	1
Widowed	0	0	5	13	2	22	1	7	1	7	9	8
**Earnings in last month**												
Nothing	0	0	2	5	0	0	0	0	1	7	3	3
1-700,000Le (USD $50)	8	22	11	29	1	11	10	67	7	50	37	33
700,000–1,000,000Le (USD $50-$70)	11	30	7	18	4	44	1	7	3	21	26	23
1,000,000–2,000,000Le (USD $70-$140)	7	19	11	29	3	33	3	20	1	7	25	22
2,000,000–3,000,000Le (USD $140-$210)	4	11	3	8	0	0	0	0	1	7	8	7
3,000,000–4,000,000Le (USD $210-$280)	2	5	2	5	0	0	0	0	0	0	4	4
4,000,000–5,000,000Le (USD $280-S350)	4	11	1	3	0	0	0	0	1	7	6	5
> 5,000,000Le (> USD $350)	0	0	1	3	0	0	1	7	0	0	2	2
Don’t know	1	3	0	0	1	11	0	0	0	0	2	2
**Years lived in fishing community (median, range)**	18 (2-47)		25 (8-62)		15 (2-52)		12 (2-28)		18 (1-33)		21 (1-62)	
**Away from community for more than 1 month at a time (in the last 12 months)**	11	30	5	13	0	0	4	27	3	21	23	20

The 17 KII participants included five staff members from MoH, one from NAS, and one from UNAIDS, as well as two staff from national fishing organizations, four members of fishermen union consortium, three site-level fishing community leaders, and one healthcare provider.

Their median age was 50 years, five (29%) were female, and participants had worked at their current organization for a median of 12 years (Table 2).

**Table 2:** Key informant interview participant demographics, N = 17.

	n	%
**Gender (% female)**	5	29
**Age (median, range); N = 15**	50 (38-66)	
**Site**		
National	9	53
Goderich	3	18
Tombo	5	29
**Type of Stakeholder/ Organization**		
Ministry of Health and Sanitation (MoHS)	5	29
National HIV and AIDS Secretariat (NAS)	1	6
UNAIDS	1	6
National fishing organization	2	12
Fishermen union consortium	4	24
Site-level fishing community leader	3	18
Healthcare provider	1	6
**Years at Organization (median, range)**	12 (4-21)	

### Awareness of HIV and HIV risk factors

All fisherfolk in the study had heard of HIV, mainly through radio/media and the community workshops and campaigns which were previously offered in their communities. Although general awareness of HIV was high, knowledge related to HIV and its risk factors was low. In the survey, only 58% and 12% correctly noted use of condoms for vaginal sex and anal sex, respectively, as a way to prevent HIV transmission, with females being more likely to note use of condoms for vaginal sex as a prevention method (p < 0.0273; RR = 1.45 (95% CI = 1.02 to 2.05)) (Table 3). Furthermore, during FGD discussions, participants had misconceptions about HIV transmission and infection, such as the belief that the virus could be transmitted by sharing toilets or that the virus causes “genitals to rot”. Many participants also recommended good hygiene and cleanliness as HIV prevention strategies.

**Table 3:** Fisherfolk participant HIV and PrEP knowledge and awareness, N = 113.

	Fishermen (N = 37)		Fishmongers (N = 38)		Fish Processors (N = 9)		Sex workers (N = 15)		Other profession (N = 14)		Females (N = 63)		Males (N = 50)		TOTAL (N = 113)		p-value	Risk Ratio (95% CI)
	n	%	n	%	n	%	n	%	n	%	n	%	n	%	n	%		
**Ways that can be used to prevent HIV transmission**																		
Use of condoms for anal sex	8	22	1	3	0	0	3	20	2	14	6	10	8	16	14	12	0.2993	
Use of condoms for vaginal sex	16	43	27	71	4	44	13	87	5	36	42	67	23	46	65	58	0.0273*	1.45 (1.02, 2.05)
Abstinence	8	22	12	32	1	11	4	27	0	0	15	24	10	20	25	22	0.6280	
PrEP	0	0	0	0	0	0	0	0	0	0	0	0	0	0	0	0	–	
PEP	0	0	0	0	0	0	0	0	0	0	0	0	0	0	0	0	–	
Reduce number of sex partners	13	35	21	55	3	33	8	53	6	43	34	54	17	34	51	45	0.0341*	1.59 (1.01, 2.49)
Being faithful to one partner	19	51	29	76	4	44	6	40	11	79	44	70	25	50	69	61	0.0317*	1.40 (1.01, 1.93)
Only have sex with people that are HIV-negative	18	49	11	29	2	22	3	20	0	0	14	22	20	40	34	30	0.0407*	0.56 (0.31, 0.99)
Don’t share sharps/needles with others	2	5	9	24	0	0	1	7	0	0	9	14	3	6	12	11	0.1556	
Don’t share other objects with others	0	0	5	13	0	0	0	0	0	0	4	6	1	2	5	4	0.3807^	
Avoid alcohol	0	0	0	0	0	0	0	0	1	7	1	2	0	0	1	1	1^	
Don’t know/declined to answer	2	5	0	0	2	22	0	0	1	7	1	2	4	8	3	3	0.1685^	
**Heard of PrEP**																	0.7308	
Yes	2	5	3	8	0	0	3	20	0	0	4	6	4	8	8	7		
No	35	95	35	92	9	100	12	80	14	100	59	94	46	92	105	93		
**How heard of PrEP (N = 8)**	N = 2		N = 3		N = 0		N = 3		N = 0		N = 4		N = 4		N = 8			
Health care providers	2	100	2	67	0	0	2	67	0	0	2	50	4	100	6	75	0.2143^	
Peer/community educator	0	0	2	67	0	0	3	100	0	0	4	100	1	25	5	63	0.1429^	
Radio or television	1	50	1	33	0	0	2	67	0	0	2	50	2	50	4	50	1^	
Non-governmental organization	0	0	0	0	0	0	2	67	0	0	2	50	0	0	2	25	0.4286^	
Support group	0	0	1	33	0	0	0	0	0	0	0	0	1	25	1	13	1^	
Internet	0	0	0	0	0	0	0	0	0	0	0	0	0	0	0	0	–	
Text message	0	0	0	0	0	0	0	0	0	0	0	0	0	0	0	0	–	
Informal conversations with others	0	0	0	0	0	0	0	0	0	0	0	0	0	0	0	0	–	
**Interested in taking PrEP** (N = 100; participants who reported being HIV negative)	N = 34		N = 32		N = 8		N = 13		N = 13		N = 54		N = 46		N = 100		0.1249	
Yes	17	50	16	50	3	38	13	100	8	62	33	61	24	52	57	57		
No	9	26	13	41	4	50	0	0	4	31	18	33	12	26	3	30		
Declined to answer	5	15	2	6	1	13	0	0	1	8	2	4	7	15	9	9		
Not applicable	3	9	1	3	0	0	0	0	0	0	1	2	3	7	4	4		

* = p-value < 0.05; ^ = Fisher’s Exact tests (all others Chi-square).

*“I have heard about it on the radio. They said that you can get it through sex, razor and shared toilet…” - Fisherman, Male, Tombo*

*“What I know of HIV is that when you have it, they said your genital parts will rot; I’m not saying [other participant] is wrong that a healthy-looking person may be a victim, but what I heard of HIV is the reality is that your genital parts will rot. But I have never seen a patient with HIV...” – Fishmonger, Female, Goderich*

KII participants were asked for their views regarding general HIV knowledge among fishing communities and to rate it on a scale of 1–10 (1 being no knowledge and 10 being a great deal of accurate knowledge). The median score was 5 (IQR: 2.75-8). Overall, KII participants agreed with the FGD and survey participants that fishing communities are aware of HIV, mainly through community education campaigns and workshops, and some participants rated HIV knowledge as high based on this general awareness. However, many stakeholders also noted that there are widespread misconceptions about HIV and that specific knowledge and understanding is low.

*“Well, I will tell you everybody in this country knows about HIV but there are some misconceptions about how HIV can be transmitted.” – KII participant, National (NAT06)*

*“Because they know HIV/AIDS is real and they know the way you get it, but if you go deep down again, they have misconceptions.” – KII participant, National (NAT06)*

More than half of the stakeholders mentioned that there are some community members who do not believe HIV exists. *“In fact, some people don’t believe that there is HIV so if you ask them to go and do test… I don’t think there is any fisherman who will volunteer to go to the hospital and say I have come to do the test.” – KII participant, Goderich (GKII01)*

Even when information is shared, many community members do not trust the information.

*“The knowledge is not there, and they don’t take the information very seriously, the concept or belief they have that HIV is not real is what is affecting them. So whatever knowledge you want to transmit to them they won’t take it seriously but view it negatively.” – KII participant, National (NAT05)*

*[They] come to the landing point… for business, money is their focus, so even when you go with HIV messages for them to sit and listen to you, you must be very tactful for them to listen to you… these people are in business time...” – KII participant, National (NAT06)*

### Awareness of community HIV prevalence

Very few fisherfolk were aware of the burden of HIV in their communities. For instance, when asked about health issues facing their communities, HIV was spontaneously mentioned by only 1 of the 113 survey participants and in only 4/12 FGDs (3 Goderich, 1 Tombo). They perceived the most common health issues amongst fisherfolk to be upper respiratory infections, tuberculosis, pneumonia, musculoskeletal pain, and malaria/fever.

*“People in this community don’t take HIV seriously like [they do] cold, malaria and other diseases, because they are afraid to expose themselves. They don’t take HIV/AIDS like that kind of deadly disease that can damage or kill.” – Fishmongers, Female, Goderich*

Several participants stated that they had never personally seen a person living with HIV, which led them to believe that HIV was not common in their communities:

*“Well, I have been in this community for long. I don’t think that HIV is a problem in this community. I have not seen someone with the signs and symptoms, so I don’t think that is a problem in this community.” – Fisherman, Male, Tombo*

*“Now that my brother here said that he has seen an HIV patient, then I am very convinced that it is true and the HIV is real in this community.” – Fisherman, Male, Tombo*

In contrast, a few respondents were aware of and concerned about HIV in the community. These were typically individuals who knew someone with HIV or had seen someone with HIV before. These participants also expressed concern that HIV was more common than people thought, and they noted that the mobility of fisherfolk made them especially vulnerable to HIV infection.

*“Well, as far as this fishing area is concerned, [it] is the headquarter for fishing, so you have a lot of people going and coming out of the community… It is not that there is no HIV, it is in the community because fisherfolks are people that travel from island to island, so there is an interaction. So, because of that, I will not deny that there is no HIV because if they are traveling to Guinea, Senegal, and Ivory Coast they have interaction with other people so things like that must happen, so because of that I will not say that there is no HIV.” – Fishmonger, Male, Tombo*

*“In this community a lot of people believe that there is no HIV. A lot of people don’t believe that HIV is real so we want the government to come with the test so that we can do the test. And I am very much convinced that if they come with the test here, a lot of people will be tested positive….” – Fisherman, Male, Tombo*

### Knowledge of HIV prevention and treatment

Many fisherfolk were aware that HIV treatment exists, that it helps people living with HIV live longer and that the medications are free.

*“Normally… when you go to the hospital they do the test, if found positive then they will counsel you and commence your treatment and this is only done at the hospital.” – Fishmonger, Female, Goderich*

*“Sometimes we see people with megaphones announce to people about HIV treatment and testing that it is free.” – Fishmonger, Female, Tombo*

In contrast to the widespread awareness of HIV treatment, only 7% of survey respondents indicated that they had ever heard of pre-exposure prophylaxis (PrEP). After being informed what PrEP was, 57% of participants who self-reported being HIV negative said they would be interested in taking PrEP (Table 3).

One KII participant mentioned PrEP in the context of inadequate access to HIV prevention services among fishing communities.

*“I don’t think we have adequate access to prevention, services like condoms like PrEP, like PEP [post-exposure prophylaxis]. I don’t think they have a lot of access to those services because they have not been adequately targeted.” – KII participant, National (NAT08)*

### Perception of HIV stigma and discrimination

In FGDs, participants noted that there is HIV stigma in the community, and some reported that they avoid people living with HIV.

*“I see some people take it very serious, because when they see the patients, they use it to gossip and discriminate them. And they do that to the relatives or main people of the patient and it is against the law to point finger at someone with HIV/AIDS.” – Fishmonger, Female, Goderich*

*“The person that I know that is positive I was not going to their compound, but the person has died* [and]*…his wife. There was also, another lady that I know who had the virus she has also died, so for me when someone has the virus, I will make sure that I don’t go to your house.” – Fishmonger, Female, Tombo*

*We hear from the jingles [radio announcements] that people that have HIV must not be discriminated against or neglected, but encourage the person and give good food in order to support them psychologically. But don’t forget to keep the rules even when you go close to the patient.” - Fisherman, Male, Goderich*

Some participants suggested quarantining people living with HIV.

*“Let the health workers go from door-to-door testing for HIV. Like what P1 said, if you have it let them take you along or invite you and put you in a separate camp until the sickness come under control.” – Fishmonger, Female, Goderich*

KII participants noted the stigma among fishing communities as well as at the health facilities that fisherfolk visit which is a barrier to accessing care.

*“...the fight against stigma and discrimination is very critical and also in the issue of educating the beneficiaries, people, their rights... because I tell you there is a lot of discrimination even coming from the health service providers within the facilities and if we don’t address those issues it’s going to affect access to services.” – KII participant, National (NAT06)*

*“The stigma is one [barrier] and then the attitude of the health care workers” – KII participant, National (NAT09)*

### HIV risk vs. risk perception among fisherfolk

**Fig 2:**
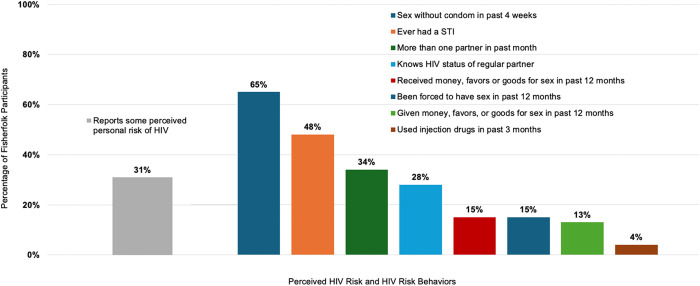
Perceived HIV risk vs. HIV risk behaviors among fisherfolk, N = 113.

Although the majority (69%) of fisherfolk survey respondents described themselves as personally being at “no or low risk” of HIV, many reported active HIV risk behaviors. When asked specifically about their sexual behavior in the survey, two-thirds (65%) reported sex without a condom in the past four weeks - with females being less likely to use a condom (p < 0.02; RR = 0.73 (95% CI = 0.56 to 0.95)), 58% were unaware of the HIV status of their regular partner, 32% had more than one partner in the past month - with males being more likely to have more than one partner than females (p < 0.0112; RR = 1.20 (95% CI = 1.15 to 3.48)) and 4% (all females) had injected drugs within the past three months (Fig 2, Table 4). Only 4% of all participants reported using lubricants when having sex within the past four weeks.

**Table 4:** Fisherfolk participant HIV risk perception and behaviors, N = 113.

	Fishermen (N = 37)		Fishmongers (N = 38)		Fish Processors (N = 9)		Sex workers (N = 15)		Other profession (N = 14)		Females (N = 63)		Males (N = 50)		TOTAL (N = 113)		p-value	Risk Ratio (95% CI)
	n	%	n	%	n	%	n	%	n	%	n	%	n	%	n	%		
**Risk Perception (Chances of getting HIV)** (N = 100; participants who reported being HIV negative)	N = 34		N = 32		N = 8		N = 13		N = 13		N = 54		N = 46		N = 100		0.2405	
No risk at all	13	38	18	56	8	100	1	8	10	77	28	52	22	48	50	50		
Small (low) risk	9	26	7	22	0	0	2	15	1	8	8	15	11	24	19	19		
Moderate risk	4	12	3	9	0	0	5	38	1	8	8	15	5	11	13	13		
Great (high) risk	7	21	1	3	0	0	3	23	0	0	4	7	7	15	11	11		
Don’t know	1	3	3	9	0	0	2	15	1	8	6	11	1	2	7	7		
**Regular partner whom you had sex in the past month**	35	95	36	95	8	89	11	73	12	86	55	87	47	94	102	90	0.3411^	
**More than one regular partner whom you had sex in the past month**	16	43	5	13	1	11	11	73	3	21	13	21	23	46	36	32	0.0112*	1.20 (1.15, 3.48)
**Know the HIV status of regular partner(s)** (N = 100; participants who reported being HIV negative)	N = 34		N = 32		N = 8		N = 13		N = 13		N = 54		N = 46		N = 100		1	
Yes	7	21	12	38	4	50	0	0	5	39	14	26	14	30	28	28		
No	24	71	15	47	4	50	9	69	6	58	29	54	29	63	58	58		
Declined to answer	1	3	1	3	0	0	0	0	0	0	1	2	1	2	2	2		
Not applicable (no regular partner)	2	6	4	13	0	0	4	31	2	15	10	19	2	4	12	12		
**Had sex without a condom in the past 4 weeks**																	0.02*	0.73 (0.56, 0.95)
Yes	29	78	22	58	6	67	9	60	7	50	35	56	38	76	73	65		
No	8	22	16	42	2	22	6	40	6	43	27	43	11	22	38	34		
Declined to answer	0	0	0	0	1	11	0	0	0	0	0	0	1	2	1	1		
Not applicable	0	0	0	0	0	0	0	0	1	7	1	2	1	2	1	1		
**Used lubricant when having sex within the past 4 weeks**																	0.0642^	
Yes	0	0	1	3	0	0	4	27	0	0	5	8	0	0	5	4		
No	37	100	36	95	8	89	11	73	13	93	56	89	49	98	105	93		
Declined to answer	0	0	1	3	1	11	0	0	0	0	1	2	1	2	2	2		
Not applicable	0	0	0	0	0	0	0	0	1	7	1	2	0	0	1	1		
**Ever received money, favors or goods for sex (past 12 months)**																	0.0005*	12.85 (1.77, 93.46)
Yes	0	0	3	8	0	0	14	93	0	0	16	25	1	2	17	15		
No	36	97	34	89	9	100	1	7	13	93	45	71	48	96	93	82		
Declined to answer	1	3	1	3	0	0	0	0	0	0	1	2	1	2	2	3		
Not applicable	0	0	0	0	0	0	0	0	1	7	1	2	0	0	1	1		
**Ever given money, favors or goods for sex (past 12 months)**																	0.4878	
Yes	7	19	1	3	0	0	7	47	0	0	7	11	8	16	15	13		
No	30	81	36	95	9	100	8	53	13	93	54	86	42	84	96	85		
Declined to answer	0	0	1	3	0	0	0	0	0	0	1	2	0	0	1	1		
Not applicable	0	0	0	0	0	0	0	0	1	7	1	2	0	0	1	1		
**Ever been forced to have sex (past 12 months)**																	0.7276	
Yes	5	14	4	11	1	11	6	40	1	7	10	16	7	14	17	15		
No	32	86	33	87	8	89	9	60	12	86	51	81	43	86	94	83		
Declined to answer	0	0	1	3	0	0	0	0	0	0	1	2	0	0	1	1		
Not applicable	0	0	0	0	0	0	0	0	1	7	1	2	0	0	1	1		
**Frequency of alcohol consumption**																	0.2703	
Never	29	78	33	87	7	78	7	47	13	93	52	83	37	74	89	79		
Monthly or less	4	11	5	13	2	22	1	7	1	7	4	6	9	18	13	12		
2-4 times a month	2	5	0	0	0	0	1	7	0	0	1	2	2	4	3	3		
2-3 times a week	1	3	0	0	0	0	3	20	0	0	3	5	1	2	4	4		
4 or more times a week	1	3	0	0	0	0	3	20	0	0	3	5	1	2	4	4		
**Consume any drugs, not including for a medical reason (past 3 months)**																	1^	
Yes	3	8	1	3	1	11	4	27	0	0	5	8	4	8	9	8		
No	34	92	37	97	8	89	11	73	14	100	58	92	46	92	104	92		
**Inject any drugs, not including for a medical reason (past 3 months)**																	0.1283^	
Yes	0	0	1	3	0	0	3	20	0	0	4	6	0	0	4	4		
No	37	100	37	97	9	100	12	80	14	100	59	94	50	100	109	96		
**Ever had a sexually transmitted infection**																	0.1387	
Yes	19	51	12	32	6	67	11	73	6	43	26	41	28	56	54	48		
No	18	49	25	66	3	33	4	27	8	57	36	57	22	44	58	51		
Don’t know/declined to answer	0	0	1	3	0	0	0	0	0	0	1	2	0	0	1	1		
**Ever been tested for HIV** (N = 100; participants who reported being HIV negative)	N = 34		N = 32		N = 8		N = 13		N = 13		N = 54		N = 46		N = 100		0.0001*	1.83 (1.29, 2.60)
Yes	13	38	29	91	5	63	11	85	6	46	44	81	20	43	64	64		
No	20	59	3	9	3	38	2	15	7	54	10	19	25	54	35	35		
Don’t know/declined to answer	1	3	0	0	0	0	0	0	0	0	0	0	1	2	1	1		
**Main reason tested for HIV** (N = 77; participants who have been tested for HIV)	N = 16		N = 35		N = 6		N = 13		N = 7		N = 53		N = 24		N = 77		0.0051	
A healthcare provider proposed to test due to sickness	5	31	15	43	6	100	4	31	1	14	22	42	9	38	31	40		
Tested as a part of an HIV testing campaign	10	63	14	40	0	0	4	31	2	29	16	30	14	58	30	39		
Pregnant	0	0	6	17	0	0	4	31	4	57	14	26	0	0	14	18		
A healthcare provider proposed to test due to being at risk	1	6	0	0	0	0	1	8	0	0	1	2	1	4	2	3		
Tested because partner is HIV-positive	0	0	0	0	0	0	0	0	0	0	0	0	0	0	0	0		
Tested when donating blood	0	0	0	0	0	0	0	0	0	0	0	0	0	0	0	0		
**Main reason never tested for HIV** (N = 35; participants who have never been tested for HIV)	N = 20		N = 3		N = 3		N = 2		N = 7		N = 10		N = 25		N = 35		0.976	
Do not feel at risk for HIV	7	35	1	33	2	67	0	0	5	71	5	50	10	40	15	43		
No time to get tested	4	20	1	33	0	0	0	0	2	29	3	30	4	16	7	20		
Stigma by healthcare providers	3	15	0	0	0	0	1	50	0	0	1	10	3	12	4	11		
Fear of positive result	2	10	0	0	0	0	1	50	0	0	1	10	2	8	3	9		
Too difficult to get testing	1	5	1	33	0	0	0	0	0	0	0	0	2	8	2	6		
Too inconvenient to get tested	1	5	0	0	1	33	0	0	0	0	0	0	2	8	2	6		
Don’t know where to get tested	1	5	0	0	0	0	0	0	0	0	0	0	1	4	1	3		
Don’t want to get tested	1	5	0	0	0	0	0	0	0	0	0	0	1	4	1	3		
Stigma by family/friends/community	0	0	0	0	0	0	0	0	0	0	0	0	0	0	0	0		
Too expensive to get tested	0	0	0	0	0	0	0	0	0	0	0	0	0	0	0	0		

* = p-value < 0.05; ^ = Fisher’s Exact tests (all others Chi-square).

Additionally, 15% of survey respondents reported receiving money and/or favors for sex in the past 12 months, 13% reported giving money and/or favors for sex in the past 12 months, and 15% reported having been forced to have sex in the past 12 months (Fig 2, Table 4). Females were more likely to report receiving money and/or favors for sex in the past 12 months (p < 0.0005; RR = 12.85 (95% CI 1.77 to 93.46)). In the FGDs, participants noted that commercial sex workers are common in fishing communities.

*“You have young girls who are commercial sex workers around and some of the fishermen have affairs with them… They hang around in the bars where they drink alcohol and exchange sex for cash.” - Fisherman, Male, Goderich*

*“There is a place here where people go and pay for girls at any price to have sex with them even if you have fifteen thousand Leone. HIV is plenty in this community, it’s just that we don’t have proof because they don’t check people.” - Fisherman, Male, Tombo*

Almost half (48%) of fisherfolks survey respondents (28 males, 26 females) reported ever having a sexually transmitted infection (STI). More males (56%) reported having an STI compared to females (41%). Within each cadre, sex workers (73%) and fish processors (67%) had the highest rates of self-reported STIs (Fig 2, Table 4). In the FGDs, syphilis and gonorrhea were mentioned as common STIs in communities.

*“We also suffer from gonorrhea and if we have sex with our partners, we may transfer the disease to them and this also can lead to the spread of HIV” - Fishing community member, Female, Goderich*

*“They do not have the knowledge that they should save in case they are ill or sick so they can have money to buy their drugs for instance STIs and STIs are not part of the free health care drugs, so they need money to buy their drugs” – KII participant, National (NAT09)*

In contrast with the reported behavior in the survey, during the FGDs, fisherfolk showed awareness that condoms and having one partner can help prevent the spread of HIV.

*“What can we do to stop the spread of HIV/AIDS considering that not everyone has a partner at home? We need to use condoms when having sex. By so doing, we can stop the spread of the virus.” - Fishmonger, Female, Goderich*

*“Well, to prevent HIV, if we want to have sex, we should make sure that we protect ourselves by using condoms.” - Fisherman, Male, Tombo*

*“They advise us to stick to one partner and that we should not have sex with other women without the use of condoms or we should avoid having sex with different women, so they gave condoms and encouraged us to stick to one partner.” Fishmonger, Male, Tombo*

### HIV testing and prevalence amongst study participants

Of the 100 fisherfolk who reported being HIV negative, only 64% had been tested for HIV. Females had higher HIV testing rates than males with 81% of females compared to 43% of males reporting ever being tested for HIV (p < 0.0001; RR = 1.83 (95% CI = 1.29 to 2.60)). The main reasons for being tested among those who reported being HIV negative and HIV positive were a healthcare provider proposed the test because the participant was sick (40%), they were tested as part of an HIV testing campaign (39%), and pregnancy (18%). Of the 35 fisherfolk who reported never being tested for HIV, the main reasons were: not feeling at risk for HIV (43%), not having time to get tested (20%), stigma from healthcare providers (11%) and fear of a positive result (9%) (Table 4).

Despite the perception that HIV was not common in the fishing community, 13 study participants (11.5%) reported that they had been diagnosed with HIV (Table 5). Only two of the 13 reported currently being on ART and both noted that it was ‘easy’ to pick up their ART medication. One reported having missed a dose in the past 7 days and both reported never having missed a dose due to being away from home or traveling.

**Table 5:** Demographics for self-reported HIV-positive participants, N = 12.

	
	N	%
**Gender (% female)**	9	69
**Age (median, range)**	44 (26-54)	
**Months since HIV diagnosis (median, range)**	24 (4-60)	
**Highest level of education**		
None	6	46
Primary	2	15
Secondary	5	38
More than secondary	0	0
**Marital status**		
Single (never married)	3	23
Married monogamous	8	62
Married polygamous	0	0
Living with partner, unmarried	0	0
Divorced/separated	0	0
Widowed	2	15
**Ever pregnant (N = 9)**	9	100
**# of living children (median, range)**	3 (1-6)	
**Primary occupation**		
Fisherman	3	23
Fishmonger	6	46
Fish processor	1	8
Sex worker	2	15
Other profession	1	8
**Currently on HIV treatment**	2	15
**Ease of picking up ART (N = 2)**		
Very difficult	0	0
Difficult	0	0
Neutral	0	0
Easy	2	100
Very easy	0	0

Of the 11 fisherfolk who reported testing HIV positive but who were not on ART, 10 said the main reason was due to feeling healthy and one said he was not taking ART due to stigma.

### HIV-specific services: access and utilization

For HIV prevention and testing services, FGD participants noted that condoms are distributed at health centers and community organizations. However, participants said that many people do not want to use condoms and that outreach condom programs had not taken place recently. Participants were unaware of any PrEP, PEP, or needle exchange services.

*“I can remember years back when they brought a sharp needle/instrument [a rapid HIV test kit] that they were using to do our HIV tests and the instant result was provided. I was part of that test, and I tested negative, I don’t know for others, because it was highly confidential. after the tests, they distributed lots of condoms. ” – Fishmonger, Female, Goderich*

Many also noted that HIV testing programs are poorly attended, and people are not always willing to get tested. Consequently, most people only get tested when they go to health facilities when they are sick.

*“The HIV/AIDS people were coming here to do free tests for the community people, the HIV people will fix megaphones all over inviting people to go and do free HIV tests, but they would never go there. And the few that would decide to go cannot go up to fifty in number. ” – Fishmonger, Female, Goderich*

*“Normally, we used to have workshop on HIV where fishing men were invited and given advice about HIV/AIDS but for the past two years that kind of thing has not been done.” – KII participant, Goderich*

One participant noted that women who are pregnant receive testing when they access care.

*“When you go to [health facility] they do TB, HIV/AIDS, and other tests before treating you and the most common way of getting people to do HIV test is when the women are pregnant it’s compulsory.” - Fishmonger, Female, Goderich*

Of the 64 fishing community members who had ever tested for HIV, not including those who reported being HIV positive, the two most common locations where they last got tested were at community health centers (63%) and through mobile outreach services (16%). On a scale of 1 (very easy) to 5 (very difficult), 51% of fisherfolk reported it was ‘easy’ or ‘very easy’ to receive HIV testing services as needed. Other respondents noted it was difficult (14%) or very difficult (2%), neutral (19%), or don’t know (14%) (Table 6).

**Table 6:** Fisherfolk participant HIV services access and utilization, N = 113.

	Fishermen (N = 37)		Fishmongers (N = 38)		Fish Processors (N = 9)		Sex workers (N = 15)		Other profession (N = 14)		Females (N = 63)		Males (N = 50)		TOTAL (N = 113)		p-value
	n	%	n	%	n	%	n	%	n	%	n	%	n	%	n	%	
**Location of last HIV test** (N = 64; participants who reported being HIV negative and having tested for HIV)	N = 13		N = 29		N = 5		N = 11		N = 6		N = 44		N = 20		N = 64		0.7921
National Referral Hospital	1	8	1	3	1	20	0	0	0	0	1	2	2	10	3	5	
District Hospital	1	8	2	7	0	0	1	9	0	0	3	7	1	5	4	6	
Community Health Center	6	46	19	66	3	60	7	64	5	83	29	66	11	55	40	63	
Community Health Post	0	0	0	0	0	0	1	9	0	0	1	2	0	0	1	2	
Maternal and Child Health Post	0	0	0	0	0	0	1	9	0	0	1	2	0	0	1	2	
Drop-in Center	0	0	0	0	0	0	0	0	0	0	0	0	0	0	0	0	
Mobile outreach services	3	23	5	17	0	0	1	9	1	17	6	14	4	20	10	16	
Through community outreach worker	0	0	0	0	0	0	0	0	0	0	0	0	0	0	0	0	
Non-governmental Organization	2	15	2	7	0	0	0	0	0	0	2	5	2	10	4	6	
Traditional health providers	0	0	0	0	0	0	0	0	0	0	0	0	0	0	0	0	
Private hospital/clinic	0	0	0	0	1	20	0	0	0	0	1	2	0	0	1	2	
**Ease of receiving HIV testing services as needed**																	
Very difficult	1	3	0	0	0	0	0	0	1	7	1	2	1	2	2	2	0.0687^
Difficult	5	14	3	8	2	22	3	20	3	21	8	13	8	16	16	14	
Neutral	9	24	7	18	1	11	3	20	2	14	10	16	12	24	22	19	
Easy	9	24	9	24	3	33	5	33	3	21	16	25	13	26	29	26	
Very easy	5	14	17	45	1	11	3	20	2	14	22	35	6	12	28	25	
Don’t know/declined to answer	8	22	2	5	2	22	1	7	3	21	6	10	10	20	16	14	
**Where do people in your community go for HIV treatment**																	
National Referral Hospital	8	22	13	34	3	33	1	7	2	14	16	25	11	22	27	24	0.5828
District Hospital	4	11	5	13	1	11	1	7	0	0	6	10	5	10	11	10	1^
Community Health Center	29	78	25	66	5	56	11	73	13	93	46	73	37	74	83	73	0.7819
Community Health Post	1	3	2	5	0	0	0	0	0	0	1	2	2	4	3	3	0.5887^
Maternal and Child Health Post	0	0	1	3	0	0	1	7	1	7	3	5	0	0	3	3	0.2496^
Drop-in Center	0	0	0	0	0	0	0	0	0	0	0	0	0	0	0	0	–
Mobile outreach services	0	0	1	3	0	0	1	7	0	0	2	3	0	0	2	2	0.4997^
Through community outreach worker	0	0	0	0	0	0	1	7	0	0	1	2	0	0	1	1	1^
Non-governmental Organization	0	0	0	0	0	0	0	0	0	0	0	0	0	0	0	0	–
Traditional health providers	0	0	0	0	0	0	0	0	0	0	0	0	0	0	0	0	–
Private hospital/clinic	0	0	0	0	0	0	0	0	0	0	0	0	0	0	0	0	–
Don’t know/declined to answer	3	8	1	3	2	22	0	0	0	0	2	3	4	8	6	5	0.4031^

^ = Fisher’s Exact tests (all others Chi-square).

For HIV treatment services, 73% of fisherfolk reported HIV treatment services are currently provided at community health centers; followed by a national referral hospital (24%) and district hospitals (10%) (Table 6).

*“Well, we get the treatment at the hospital. After when they test you if you are positive, they start giving you the treatment and sometimes they refer them.” – Fishmonger, Females, Tombo*

In the FGDs, some participants stated that they were not aware of any HIV services available in the health facilities near their community to people living with HIV.

*“Well, I don’t know if a HIV patient presents to the health facility if they are treated. As far as I know… there is no health facility in the community that is dedicated for HIV.” - Fishing community member, Male, Goderich*

### HIV services preferences

**Fig 3:**
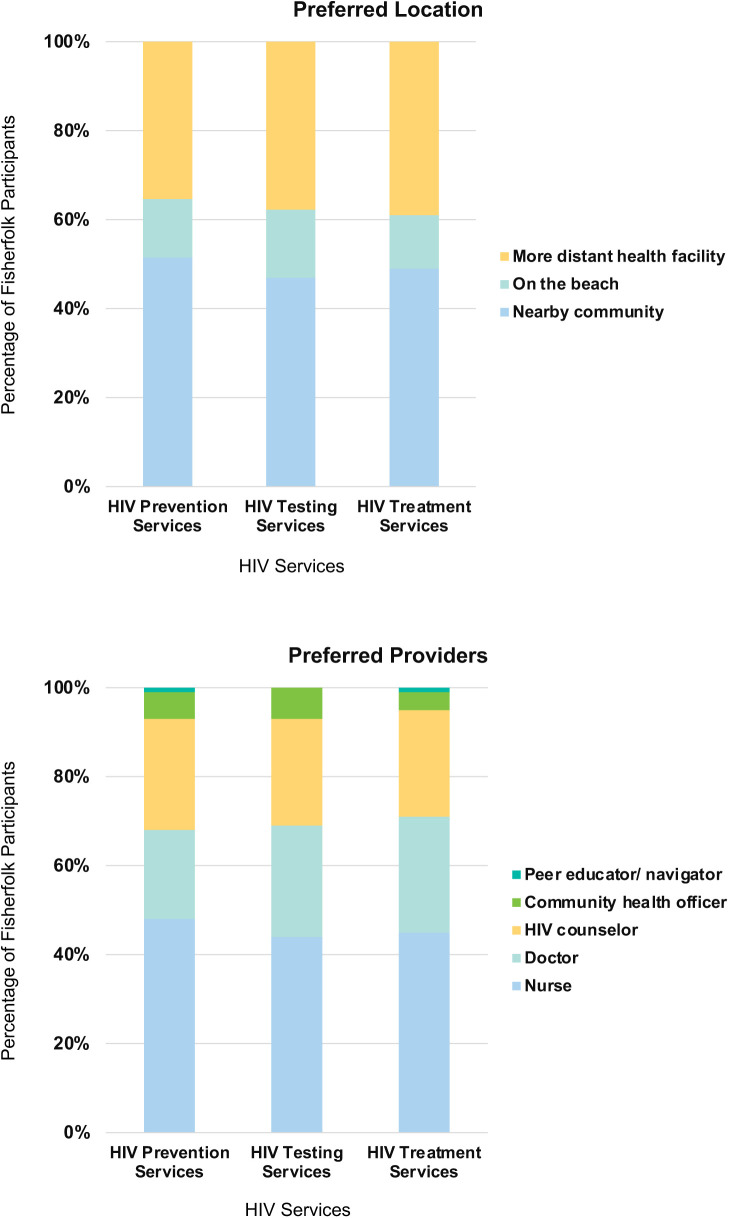
Preferred location and providers for HIV services among fisherfolk, N = 113.

More survey respondents preferred to receive HIV prevention, testing, and treatment services in the nearby community (versus on the beach or at more distant health facilities) and from nurses (versus doctors, HIV counselors, community health officers or other cadres) (Fig 3).

For HIV prevention services in particular, fisherfolk agreed that community sensitization and education is key to supporting HIV prevention, explaining that the community needs to know more about HIV in order to take action to prevent it. Fisherfolk preferred that services come to the community to provide prevention education and supply condoms.

*“I think the community sensitization is the best way that we can protect the spread of the virus because if you tell someone what they should do to prevent themselves, that is the best way.” – Fisherman, Male, Tombo*

*“What I know is they do provide testing services, but [it] is not compulsory it’s voluntary and, in most cases, when they asked them to do an HIV test, they reject it. So it’s difficult to know the percentage of HIV cases in these communities, so what we are actually looking at is, if we can get an isolated area where they can secretly go and do tests and it should be highly confidential.” – KII participant, MFMR*

Fisherfolk viewed the availability of HIV testing services at government hospitals and community facilities as generally sufficient. However, many participants emphasized the importance of appropriate counseling and motivational support to address the fear and stigma associated with HIV testing within their communities.

Regarding HIV treatment services, fisherfolk underscored the necessity for healthcare providers to undergo specialized training and sensitization in order to deliver care tailored to the unique needs of the fisherfolk community. Additionally, there was a widespread call among fisherfolk for the establishment of a dedicated health center within their community. This facility would not only provide HIV-related services but also include broader healthcare services to enhance access to all healthcare needs.

Key stakeholders echoed these concerns, advocating for greater recognition of fisherfolk as a priority population in Sierra Leone’s HIV response efforts.

*“We know the fisherfolks are a vulnerable population but they are not given that prominence. And these are the people that we really need to focus on in terms of HIV because [they are] a mobile population… mobile in the sense that somebody can fish today in Sierra Leone waters and let me say that person is residing at Goderich, that person might decide to go and land the catch in [a far away location] and that person can stay there for a longer period. That person doesn’t only have like one base, whilst if you’re a businessman and you have your shop here that’s where they know you, but they are always on the move so I consider them as mobile population and also people who can afford to pay for sex…” – KII participant, NAS*

*“Well, it is to bring in more health centers so that those centers will help the fishermen.” – KII participant, Goderich*

*“I think we are part… we are the policymakers and programme implementers because we haven’t consciously targeted them with HIV services to support treatment care services, [that is] the [HIV treatment] cascade. We haven’t consciously targeted them as a deliberate policy to reach out to them. There was no engagement and that’s what I think is lacking.” – KII participant, UNAIDS*

## Discussion

Globally, fisherfolk are at high risk for HIV compared to the general population [2,3,9]. This study provides insights into HIV knowledge, attitudes and behaviors and HIV service preferences among fisherfolk in Sierra Leone. Despite awareness of HIV and HIV services, mainly driven by past community education campaigns, critical knowledge gaps persist, particularly regarding modes of transmission and prevention methods. Many participants reported misconceptions and inaccurate beliefs about how HIV is transmitted, which is consistent with a study in Uganda which also observed HIV knowledge gaps related to modes of transmission and found that only 51% of fisherfolk had comprehensive knowledge of HIV prevention [29].

### HIV Risk: Perceptions and behaviors

Perceptions of HIV risk among fisherfolk in Sierra Leone were generally low, with 69% of survey respondents describing themselves as being at “no” or “low risk” of HIV. Despite believing themselves to be at low risk of HIV, however, many fisherfolk reported high-risk behaviors such as having had sex without a condom in the past four weeks (65%), having more than one partner in the past month (32%), and participating in transactional sex (15% receiving and 13% giving money and/or favors in exchange for sex) in the past 12 months. Males were less likely to know the HIV status of their regular partners (35% compared to 46%) and to have more than one regular sexual partner in the past four weeks (46% males, 21% females, p < 0.0112), although 73% of sex workers (all female) had more than one regular sexual partner. Males were also significantly more likely to report using a condom in the past four weeks (76% males, 56% females, p < 0.02). Use of lubricants was very rare with only 5 participants, all females, reporting using lubricants in the past four weeks.

These rates of high-risk behaviors are consistent with many other studies among fisherfolk. In the 2010 Sierra Leone HIV Modes of Transmission Study 63% of fisherfolk reported never using a condom with non-regular partners [21]. Inconsistent condom use among fisherfolk is universally high as reported in many studies in a variety of fishing communities: 40% in Ghana, 48% in Tanzania, and 95% in Uganda [30,31,32].

Exchanging sex for money or goods is common in fishing communities where the division of labor is highly gendered, with men primarily responsible for catching fish and women handling processing and trade. The roles and power dynamics in the community are conducive to transactional sex [2,33]. Declines in fish can lead to higher rates of transactional sex due to food insecurity and economic challenges [33,34]. Of note, in our study, sex workers reported both giving and receiving money or goods in exchange for sex, which is an unusual pattern in the context of transactional sex.

Almost half (48%) of the survey participants reported ever having an STI. A study among fishermen in Kisumu, Kenya reported that 90% of fishermen had evidence of one or more STI [12]. Our study observed lower rates of drug and alcohol use compared to other studies [13,35]. This may be due to the fact that 77% of the population of Sierra Leone is Muslim, compared to lower rates in the East African countries studied [36].

In our study, self-reported HIV prevalence among fisherfolk was 12%, with 13 individuals reporting being HIV-positive. This prevalence rate is much higher than the 3.9% reported in the 2011 HIV Surveillance on Fisherfolks in Sierra Leone report [20], but similar to studies in other countries, which found, for example, 11.3% among fishermen [37] and 14% among fisherfolk [31] in Tanzania and 10% among fisherfolk in Uganda [15]. Higher rates were found in other studies among fishing communities in Uganda at 22–40% [16] and Kenya at 32% [38]. In our study, only 2 (15%) of the individuals self-reporting being HIV-positive were currently receiving HIV treatment, which is much lower than reported in two studies in Tanzania with rates of current HIV treatment of 64% and 78% [31,37]. This may be due to self-reporting and barriers such as low social support, lack of access to healthcare, mobility, and stigma [15], or to misreporting of HIV status.

### HIV services: Availability, utilization and preferences

Access to and utilization of HIV prevention, testing, and treatment services among fisherfolk in Sierra Leone were mixed, with notable barriers and gaps in service delivery. For HIV prevention, participants were aware that condoms are distributed at health centers and by community organizations. Some participants recalled past HIV testing campaigns that included condom distribution but indicated that such initiatives had not occurred in recent years. Additionally, there was a lack of awareness of PrEP as a preventive option, with only 8% of fisherfolk ever hearing of PrEP. This highlights a major gap among a vulnerable population who have a low perceived risk of HIV, yet high-risk behaviors. Raising awareness of HIV risk and PrEP and improving access to PrEP could support greater PrEP uptake among HIV-negative fisherfolk [39,40].

HIV testing services were described as poorly attended, with many individuals reluctant to participate. Among the 100 fisherfolk who self-reported as HIV-negative in our study, only 64% reported ever being tested for HIV. This rate was much higher than the 10% ever having tested in Ghana [30] and more aligned with results in Tanzania where 61% and 77% of fisherfolk had tested for HIV in the past 12 months [37,31]. Participants noted that most community members only get tested when they are already visiting health facilities due to illness. It is important to note that in Sierra Leone pregnant women are required to be tested for HIV [41], however, this requires pregnant women to access healthcare services, and as of 2023, 23% of deliveries took place at home [42]. Of study participants who reported ever testing for HIV, the majority (63%) were last tested at community health centers, while 16% utilized mobile outreach services. This highlights the need and opportunity for reaching fisherfolk where they are through more community outreach and targeted testing campaigns. HIV self-testing, especially through peer networks, has been shown to be effective in engaging hard to reach populations [43,44] including fisherfolk [45,46,47]. HIV self-testing was not a focus of this study.

Fisherfolk had varying levels of awareness of how to access HIV treatment within their community, with some not being aware of any treatment services within their community. Inconsistent outreach efforts and low engagement pose challenges to effective HIV services.

Fisherfolk expressed preference for receiving HIV prevention, testing, and treatment services at health facilities within their communities rather than at the beach or distant health facilities. They wanted HIV and general health services brought to their community, including sensitization and education. Many participants advocated for establishing a dedicated health center in their communities to provide HIV services alongside broader healthcare needs. In our study, fisherfolk expressed high demand and desire for health education and integrated services in their communities.

In 2018, MoH in Sierra Leone published guidelines for HIV differentiated service delivery (DSD) recognizing the need to tailor HIV services for specific communities [23]. Preferences for HIV services among fisherfolk emphasize the importance of accessibility, tailored care, and community-centered approaches, with significant opportunities to incorporate DSD to better meet their needs. Fishing communities are distinct and dynamic, characterized by interconnected social networks, central landing sites serving as hubs, and highly mobile fishermen. To effectively support their work and lifestyle, healthcare services must be flexible and accessible [48]. Utilizing peers to provide community-based services could support access to and uptake of HIV and general health services among fisherfolk, a strategy that has been proven effective for other vulnerable and mobile populations [46,47,49,50]. Given their mobility, DSD for fisherfolk should include multi-month dispensing of HIV and other medications, mobile health, and decentralization, and integrated services [51]. These flexible and person-centered services could further support HIV service delivery access, uptake, and treatment adherence among fisherfolk. It is also essential to involve fisherfolk in the design, delivery, and evaluation of HIV services provided to their community [52].

Key informants and stakeholders emphasized the need to adapt HIV services to the mobility patterns of fisherfolk, integrating flexible and context-specific differentiated service delivery models into national HIV response strategies. Stakeholders also noted that deliberate policy efforts to recognize fisherfolk as a priority population would ensure that services are designed to meet their unique needs and circumstances. Incorporating differentiated service delivery into HIV prevention, testing, and treatment strategies offers a promising opportunity to improve access, reduce stigma, and enhance the overall effectiveness of HIV services for fisherfolk communities.

### Study limitations

This study has several limitations. First, it was conducted in a small purposively-selected sample of two fishing communities in high HIV-prevalence districts of Sierra Leone, and findings may not be generalizable to all fisherfolk or fishing communities in the country or other settings. The study also relied on a convenience sample of fisherfolk who were available and willing to participate, which may introduce selection bias.

The cross-sectional nature of the study also limits its ability to draw conclusions about causal relationships or changes over time. Funding constraints prevented the inclusion of more diverse data collection approaches, such as longitudinal studies or participatory research methods, which could have enhanced the understanding of fisherfolk’s healthcare needs and preferences.

Furthermore, HIV status was based solely on self-report, without confirmatory HIV testing. This reliance on self-reported data may result in inaccuracies due to misreporting of HIV status, which could affect the interpretation of findings related to HIV prevalence, treatment access, and service preferences.

Future research should consider longitudinal and mixed-methods approaches that include a larger and more representative sample, as well as specific strategies to address the challenges associated with studying mobile and hard-to-reach populations.

## Conclusions

Fisherfolk reported limited awareness of HIV prevention strategies, relatively high levels of HIV risk behaviors, and stigma and discrimination towards people living with HIV. Although current HIV prevalence amongst fisherfolk in Sierra Leone is unknown, 12% of participants in this small study reported having been diagnosed with HIV. Fisherfolk are eager to receive more healthcare services in general and more HIV services specifically. They are clear about healthcare barriers such as mobility, cost, and perceived low-quality public sector health services, and open to participating in the optimal design of HIV service delivery. Working with fishing communities to design DSD strategies will be an important part of expanding access to HIV testing services, linkage to prevention, including PrEP, for those testing negative, and linkage to treatment for those testing positive.

## Supporting information

S1 Checklist PLOS ‘Inclusivity in global research’ form. (DOCX)

## References

[1] Kwena ZA, Camlin CS, Shisanya CA, Mwanzo I, Bukusi EA. Short-term mobility and the risk of HIV infection among married couples in the fishing communities along Lake Victoria, Kenya. PLoS One. 2013;8(1):e54523. doi: 10.1371/journal.pone.0054523 23336005 PMC3545885

[2] Kissling E, Allison EH, Seeley JA, Russell S, Bachmann M, Musgrave SD, et al. Fisherfolk are among groups most at risk of HIV: cross-country analysis of prevalence and numbers infected. AIDS. 2005;19(17):1939–46. doi: 10.1097/01.aids.0000191925.54679.94 16260899

[3] Seeley J, Nakiyingi-Miiro J, Kamali A, Mpendo J, Asiki G, Abaasa A, et al. High HIV incidence and socio-behavioral risk patterns in fishing communities on the shores of Lake Victoria, Uganda. Sex Transm Dis. 2012;39(6):433–9. doi: 10.1097/OLQ.0b013e318251555d 22592828

[4] Malaysian AIDS Council. No such thing as calm: A policy brief on fishermen, HIV and human rights. 2016. https://www.aidsdatahub.org/resource/no-such-thing-calm-policy-brief-fishermen-hiv-and-human-rights

[5] Wanyonyi IN, Wamukota A, Mesaki S, Guissamulo AT, Ochiewo J. Artisanal fisher migration patterns in coastal East Africa. Ocean & Coastal Management. 2016;119:93–108. doi: 10.1016/j.ocecoaman.2015.09.006

[6] Cinner JE, McClanahan TR, Wamukota A. Differences in livelihoods, socioeconomic characteristics, and knowledge about the sea between fishers and non-fishers living near and far from marine parks on the Kenyan coast. Marine Policy. 2010;34(1):22–8. doi: 10.1016/j.marpol.2009.04.003

[7] Nunan F. Mobility and fisherfolk livelihoods on Lake Victoria: Implications for vulnerability and risk. Geoforum. 2010;41(5):776–85. doi: 10.1016/j.geoforum.2010.04.009

[8] Njock J-C, Westlund L. Migration, resource management and global change: Experiences from fishing communities in West and Central Africa. Marine Policy. 2010;34(4):752–60. doi: 10.1016/j.marpol.2010.01.020

[9] Allison EH, Seeley JA. HIV and AIDS among fisherfolk: a threat to ‘responsible fisheries’?. Fish and Fisheries. 2004;5(3):215–34. doi: 10.1111/j.1467-2679.2004.00153.x

[10] Nakamanya S, Okello ES, Kwena ZA, Nanyonjo G, Bahemuka UM, Kibengo FM, et al. Social networks, mobility, and HIV risk among women in the fishing communities of Lake Victoria. BMC Womens Health. 2022;22(1):555. doi: 10.1186/s12905-022-02144-8 36578062 PMC9798550

[11] Tumwesigye NM, Atuyambe L, Wanyenze RK, Kibira SP, Li Q, Wabwire-Mangen F, et al. Alcohol consumption and risky sexual behaviour in the fishing communities: evidence from two fish landing sites on Lake Victoria in Uganda. BMC Public Health. 2012;12:1069. doi: 10.1186/1471-2458-12-1069 23231779 PMC3534008

[12] Kwena ZA, Bukusi EA, Ng’ayo MO, Buffardi AL, Nguti R, Richardson B, et al. Prevalence and risk factors for sexually transmitted infections in a high-risk occupational group: the case of fishermen along Lake Victoria in Kisumu, Kenya. Int J STD AIDS. 2010;21(10):708–13. doi: 10.1258/ijsa.2010.010160 21139150

[13] Sileo KM, Kizito W, Wanyenze RK, Chemusto H, Reed E, Stockman JK, et al. Substance use and its effect on antiretroviral treatment adherence among male fisherfolk living with HIV/AIDS in Uganda. PLoS One. 2019;14(6):e0216892. doi: 10.1371/journal.pone.0216892 31158232 PMC6546219

[14] Toms K, Potter H, Balaba M, Parkes-Ratanshi R. Efficacy of HIV interventions in African fishing communities: A systematic review and qualitative synthesis. Int J Infect Dis. 2020;101:326–33. doi: 10.1016/j.ijid.2020.09.1476 33017696

[15] Bogart LM, Naigino R, Maistrellis E, Wagner GJ, Musoke W, Mukasa B, et al. Barriers to Linkage to HIV Care in Ugandan Fisherfolk Communities: A Qualitative Analysis. AIDS Behav. 2016;20(10):2464–76. doi: 10.1007/s10461-016-1331-z 26961380 PMC5507699

[16] Musumari PM, Techasrivichien T, Srithanaviboonchai K, Wanyenze RK, Matovu JKB, Poudyal H, et al. HIV epidemic in fishing communities in Uganda: A scoping review. PLoS One. 2021;16(4):e0249465. doi: 10.1371/journal.pone.0249465 33793652 PMC8016276

[17] Food and Agriculture Organization (FAO) of the United Nations. Fishery and Aquaculture Country Profiles. Sierra Leone. 2024. https://www.fao.org/fishery/en/facp/sle?lang=en

[18] Kawuki J, Kamara K, Sserwanja Q. Prevalence of risk factors for human immunodeficiency virus among women of reproductive age in Sierra Leone: a 2019 nationwide survey. BMC Infect Dis. 2022;22(1):60. doi: 10.1186/s12879-022-07037-7 35039011 PMC8764866

[19] Julius OO, Conteh ZF. Benefit Related Relationships among Artisanal Fisher Folks of Tombo Fish Landing Community Western Sierra Leone. J of Fisheries and Aquatic Science. 2014;9(5):387–97. doi: 10.3923/jfas.2014.387.397

[20] National HIV/AIDS Secretariat (NAS). HIV Surveillance Among Fisherfolks in Sierra Leone. 2011. https://www.nas.gov.sl/publication/109-hiv-surveillance-among-fisherfolks-in-sierra-leone

[21] National HIV/AIDS Secretariat (NAS). Sierra Leone HIV Modes of Transmission Study - Know Your Epidemic, Know Your Response. 2010. https://www.nas.gov.sl/publication/99-sierra-leone-hiv-modes-of-transmission-study

[22] National HIV/AIDS Secretariat (NAS). National Strategic Plan on HIV/AIDS 2016-2020. 2025. https://portal.mohs.gov.sl/wp-content/uploads/2021/04/sierra-leone-hiv-national-strategic-plan-2016-2020.pdf

[23] Ministry of Health and Sanitation SL. Guide of Differentiated care model in Sierra Leone: Who feels it knows it. 2018. https://www.differentiatedservicedelivery.org/wp-content/uploads/Guide-of-Differentiated-care-Model-Sierra-Leone-Final-Version-May-2018-1.pdf

[24] Overview: A matter of choice: people and possibilities in the age of AI. Human Development Report. United Nations. 2025. p. 1–11. doi: 10.18356/9789211542639c003

[25] UNAIDS. Sierra Leone Country Factsheet. https://www.unaids.org/en/regionscountries/countries/sierraleone. Accessed 2023.

[26] Statistics Sierra Leone SSL, ICF. Sierra Leone Demographic and Health Survey 2019. 2020. https://dhsprogram.com/publications/publication-FR365-DHS-Final-Reports.cfm

[27] Ritchie J, Lewis J. Qualitative research practice: a guide for social science students and researchers. London: Sage. 2003.

[28] Garrison DR, Cleveland-Innes M, Koole M, Kappelman J. Revisiting methodological issues in transcript analysis: Negotiated coding and reliability. The Internet and Higher Education. 2006;9(1):1–8. doi: 10.1016/j.iheduc.2005.11.001

[29] Kwagonza L, Bulage L, Okello PE, Kusiima J, Kadobera D, Ario AR. Comprehensive knowledge of HIV prevention among fishing communities of Lake Kyoga, Uganda, 2013. BMC Public Health. 2020;20(1):29. doi: 10.1186/s12889-020-8146-6 31914966 PMC6950879

[30] Kyei-Gyamfi S. Factors affecting condom use among fishers in Elmina fishing community in Ghana. J Public Health Res. 2023;12(3):22799036231191035. doi: 10.1177/22799036231191035 37655294 PMC10467189

[31] Kapesa A, Basinda N, Nyanza EC, Mushi MF, Jahanpour O, Ngallaba SE. Prevalence of HIV infection and uptake of HIV/AIDS services among fisherfolk in landing Islands of Lake Victoria, north western Tanzania. BMC Health Serv Res. 2018;18(1):980. doi: 10.1186/s12913-018-3784-4 30563534 PMC6299499

[32] Burgos-Soto J, Ben Farhat J, Alley I, Ojuka P, Mulogo E, Kise-Sete T, et al. HIV epidemic and cascade of care in 12 east African rural fishing communities: results from a population-based survey in Uganda. BMC Public Health. 2020;20(1):970. doi: 10.1186/s12889-020-09121-6 32560717 PMC7305611

[33] Oswald G. Baiting the hook: Fish scarcity, gendered division of labour, and the fish-for-sex trade. Soc Sci Med. 2024;345:116594. doi: 10.1016/j.socscimed.2024.116594 38382334

[34] Fiorella KJ, Camlin CS, Salmen CR, Omondi R, Hickey MD, Omollo DO, et al. Transactional Fish-for-Sex Relationships Amid Declining Fish Access in Kenya. World Development. 2015;74:323–32. doi: 10.1016/j.worlddev.2015.05.015

[35] Kuteesa MO, Weiss HA, Cook S, Seeley J, Ssentongo JN, Kizindo R, et al. Epidemiology of Alcohol Misuse and Illicit Drug Use Among Young People Aged 15-24 Years in Fishing Communities in Uganda. Int J Environ Res Public Health. 2020;17(7):2401. doi: 10.3390/ijerph17072401 32244722 PMC7178227

[36] U.S. Department of State. 2023 Report on International Religious Freedom: Sierra Leone. 2023. https://www.state.gov/reports/2023-report-on-international-religious-freedom/sierra-leone/

[37] Mwanahapa P, Mtoro MJ, Gerald D, Horumpende P, Mujeeb S. Prevalence of HIV infection and uptake of HIV/AIDs services among fishermen on the shores of Lake Victoria in Kagera region, Northwestern Tanzania. PLoS One. 2025;20(1):e0315265. doi: 10.1371/journal.pone.0315265 39854333 PMC11760556

[38] Kwena ZA, Njuguna SW, Ssetala A, Seeley J, Nielsen L, De Bont J, et al. HIV prevalence, spatial distribution and risk factors for HIV infection in the Kenyan fishing communities of Lake Victoria. PLoS One. 2019;14(3):e0214360. doi: 10.1371/journal.pone.0214360 30908555 PMC6433243

[39] Ntabadde K, Kagaayi J, Ssempijja V, Feng X, Kairania R, Lubwama J, et al. Pre-exposure prophylaxis (PrEP) knowledge, use, and discontinuation among Lake Victoria fisherfolk in Uganda: a cross-sectional population-based study. medRxiv. 2024;:2024.03.29.24305076. doi: 10.1101/2024.03.29.24305076 40343955 PMC12063894

[40] Bogart LM, Musoke W, Mukama CS, Allupo S, Klein DJ, Sejjemba A, et al. Enhanced Oral Pre-exposure Prophylaxis (PrEP) Implementation for Ugandan Fisherfolk: Pilot Intervention Outcomes. AIDS Behav. 2024;28(10):3512–24. doi: 10.1007/s10461-024-04432-w 39028385 PMC11427177

[41] UNAIDS. Country progress report - Sierra Leone. 2020. https://www.unaids.org/sites/default/files/country/documents/SLE_2020_countryreport.pdf

[42] UNICEF. UNICEF in Sierra Leone: Transforming lives and building resilience: 2023 Annual Report. 2023. www.unicef.org/sierraleone/reports/unicef-sierra-leone-2023-annual-report

[43] Tucker JD, Wei C, Pendse R, Lo Y-R. HIV self-testing among key populations: an implementation science approach to evaluating self-testing. J Virus Erad. 2015;1(1):38–42. doi: 10.1016/S2055-6640(20)31145-6 26005717 PMC4439005

[44] Majam M, Hatzold K, Mavhu W, Tembo A, Zishiri V, Phiri J, et al. Reaching priority populations with different HIV self-testing distribution models in South Africa: an analysis of programme data. BMC Infect Dis. 2025;22(Suppl 1):981. doi: 10.1186/s12879-025-10662-7 40001039 PMC11863391

[45] Matovu JKB, Nambuusi A, Wanyenze RK, Serwadda D. Peer-leaders’ experiences and challenges in distributing HIV self-test kits in a rural fishing community, Rakai, Uganda. BMC Public Health. 2021;21(1):708. doi: 10.1186/s12889-021-10804-x 33845811 PMC8042983

[46] Lewis-Kulzer J, Olugo P, Gutin SA, Kwena ZA, Nishimura H, Thorp M, et al. “There is no need to leave the beach to test”: a qualitative study of HIV self-testing knowledge and acceptability of HIV self-test kit distribution among social networks of fishermen in Western Kenya. BMC Public Health. 2025;25(1):1005. doi: 10.1186/s12889-025-22136-1 40087617 PMC11908045

[47] Camlin CS, Sheira LA, Kwena ZA, Charlebois ED, Agot K, Moody J, et al. The effect of a social network-based intervention to promote HIV testing and linkage to HIV services among fishermen in Kenya: a cluster-randomised trial. Lancet Glob Health. 2025;13(4):e669–78. doi: 10.1016/S2214-109X(24)00539-4 40155104 PMC12431777

[48] Camlin CS, Cassels S, Seeley J. Bringing population mobility into focus to achieve HIV prevention goals. J Int AIDS Soc. 2018;21 Suppl 4(Suppl Suppl 4):e25136. doi: 10.1002/jia2.25136 30027588 PMC6053544

[49] Lindsay BR, Mwango L, Toeque M-G, Malupande SL, Nkhuwa E, Moonga CN, et al. Peer community health workers improve HIV testing and ART linkage among key populations in Zambia: retrospective observational results from the Z-CHECK project, 2019-2020. J Int AIDS Soc. 2022;25(11):e26030. doi: 10.1002/jia2.26030 36317821 PMC9624072

[50] Bernays S, Tshuma M, Willis N, Mvududu K, Chikeya A, Mufuka J, et al. Scaling up peer-led community-based differentiated support for adolescents living with HIV: keeping the needs of youth peer supporters in mind to sustain success. J Int AIDS Soc. 2020;23 Suppl 5(Suppl 5):e25570. doi: 10.1002/jia2.25570 32869532 PMC7459167

[51] Thorp M, Ayieko J, Hoffman RM, Balakasi K, Camlin CS, Dovel K. Mobility and HIV care engagement: a research agenda. J Int AIDS Soc. 2023;26(3):e26058. doi: 10.1002/jia2.26058 36943731 PMC10029995

[52] ITPC Global. Community Engagement Framework for Differentiated Service Delivery. 2022. https://itpcglobal.org/resource/community-engagement-framework-for-differentiated-service-delivery/

